# Case Report: Intravenous fosphenytoin successfully treated acute exacerbation of secondary trigeminal neuralgia due to petroclival meningioma

**DOI:** 10.3389/fpain.2026.1817373

**Published:** 2026-04-22

**Authors:** Yoshiki Mochizuki, Masahito Kobayashi, Sachiko Hirata, Kazuhiko Takabatake, Yutaka Mine, Takamitsu Fujimaki

**Affiliations:** Department of Neurosurgery, Saitama Medical University, Moroyama-machi, Japan

**Keywords:** fosphenytoin, petroclival meningioma, secondary trigeminal neuralgia, sodium channel blocker, trigeminal neuralgia crisis

## Abstract

**Introduction:**

Trigeminal neuralgia (TN) is a severe paroxysmal neuropathic pain disorder. While various strategies have been proposed for managing acute exacerbations of classical TN, treatment approaches for secondary TN remain largely undocumented. Here, to the best of our knowledge, we present the first reported case of secondary TN crisis due to a petroclival meningioma, successfully managed with intravenous fosphenytoin.

**Case description:**

A 62-year-old woman developed right-sided TN secondary to a petroclival meningioma. Her facial pain resolved after tumor resection but recurred six years later with slight tumor regrowth. Despite escalation of carbamazepine (CBZ) and CyberKnife radiosurgery, she experienced a TN crisis, presenting with persistent severe pain and impaired oral intake. Intravenous fosphenytoin was administered at loading dose over 20 min, resulting in nearly complete pain relief. Because of symptom recurrence, fosphenytoin was continued at maintenance dose for seven days, in parallel with CBZ titration to 800 mg/day and addition of baclofen. Oral intake improved rapidly thereafter, and no further acute exacerbations were observed over three months of follow-up.

**Conclusion:**

This case highlights the potential of intravenous fosphenytoin as an effective and rapid treatment for acute crisis of secondary TN, especially when oral therapy is not feasible.

## Introduction

Trigeminal neuralgia (TN) is a debilitating paroxysmal neuropathic pain disorder localized to the distribution of the trigeminal nerve. Despite adequate pharmacological treatment, patients may still experience intolerable pain, occasionally escalating to severe acute exacerbations that impair oral intake and necessitate hospitalization. In the literature, these episodes have been referred to as “acute exacerbations” or “acute flare-ups” of TN (1, 2), “status trigeminal neuralgia” (3) or “trigeminal neuralgia crisis” (4, 5). In recent years, intravenous fosphenytoin has been reported to be effective for management of acute TN exacerbations, though exclusively for classical TN (5–9). Here we document the potential efficacy of intravenous fosphenytoin for TN crisis secondary to petroclival meningioma.

## Case description

**Figure 1:**
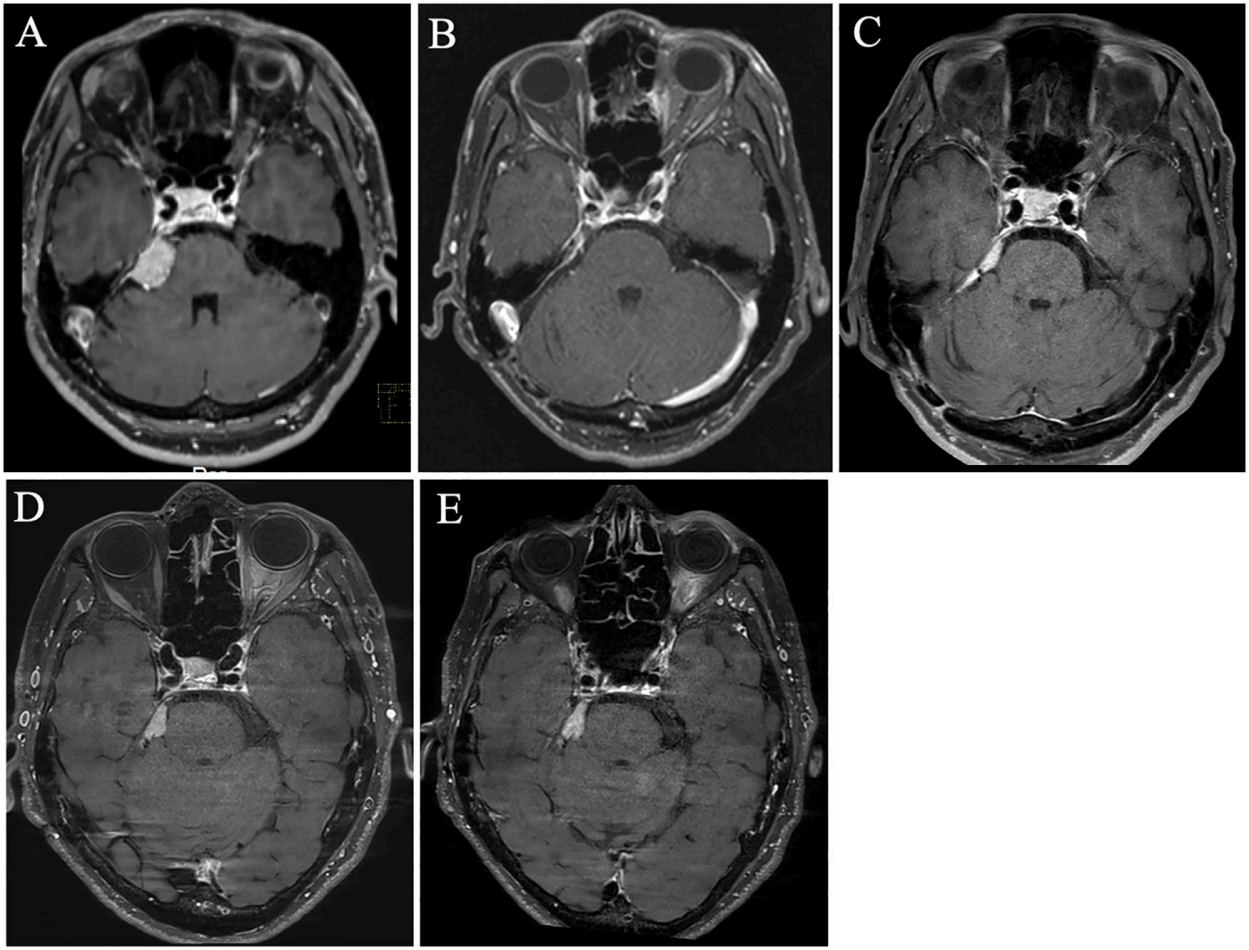
**(A)** Gadolinum-enhanced T1-weighted magnetic resonance imaging (MRI) at initial presentation shows a right petroclival meningioma. **(B)** Postoperative MRI demonstrates no residual tumor in the cerebellopontine angle. **(C)** Follow-up MRI at three years after surgery reveals slight tumor regrowth. **(D)** MRI 8 years after surgery, prior to Cyber Knife treatment, shows gradual tumor progression around the trigeminal nerve. **(E)** No significant tumor progression is evident on MRI at the time of the trigeminal neuralgia crisis, when compared to Figure D.

A 62-year-old woman initially presented to our hospital with severe numbness and pain in the right oral cavity that developed following facial washing. Magnetic resonance imaging (MRI) revealed a right petroclival meningioma (Figure 1A). Seven months after initial presentation, she underwent tumor resection via craniotomy. Intraoperatively, the tumor was found to be firmly adherent to cranial nerves Ⅴ, Ⅶ, and Ⅷ, all of which were carefully dissected and preserved. Pathological examination confirmed an angiomatous meningioma, CNS WHO grade 1 (10). Postoperatively, her facial pain resolved and no medication was required (Figure 1B).

Six years after surgery, her facial pain recurred, characterized by electric shock-like sensations triggered by speaking, along with persistent paresthesia. Carbamazepine (CBZ) was initiated, and her symptoms gradually improved with dose escalation. However, at seven years postoperatively, gadolinium-enhanced T1-weighted MRI revealed slight tumor regrowth with contact to the brainstem when compared with images obtained three years earlier (Figure 1C). Her pain worsened, particularly during eating and speaking. The CBZ dose was increased, but this led to gastrointestinal discomfort, and therefore the patient declined any further escalation.

Eight years postoperatively, in response to progressive TN symptoms and radiographic evidence of tumor growth, CyberKnife radiosurgery was performed (Figure 1D). Despite an increase of CBZ to 300 mg/day, the facial pain worsened, being initially triggered by even gentle touching of the face, but later occurred spontaneously, each episode lasting for several tens of seconds. The patient suffered persistent pain classed as 5 on the numerical rating scale (NRS), with paroxysmal exacerbations reaching NRS 9. Oral intake became severely impaired, leading to a diagnosis of TN crisis. No tumor growth was observed on MRI at that time (Figure 1E).

The patient then received an intravenous infusion of fosphenytoin at a dose of 18 mg/kg over 20 min, resulting in almost complete pain relief, with the NRS score decreasing to 1 within 20 min. However, symptoms recurred on the following day, necessitating continued administration of fosphenytoin at 9.0 mg/kg. In parallel, CBZ was gradually titrated to 800 mg/day over approximately one week, and baclofen was added at a dose of 10 mg/day. Fosphenytoin was administered for a total of seven days, and the patient regained her oral intake. No adverse effects were observed. During maintenance therapy, the pain remained controlled at approximately NRS 1–3. Since the final treatment, no further acute exacerbation of TN has occurred for three months.

## Discussion

We have described a case of secondary TN due to a petroclival meningioma in which acute exacerbation occurred but showed a dramatically favorable response to intravenous administration of fosphenytoin. To our knowledge, this is the first report to describe the effectiveness of intravenous fosphenytoin as an acute-phase treatment for secondary TN crisis. Fosphenytoin is a prodrug of phenytoin. In comparison to conventional phenytoin injection, it has a reduced risk of vascular pain and phlebitis and can be administered in a shorter time to treat status epilepticus. The use of fosphenytoin for treatment of acute exacerbation of classical TN has already been reported, as well as a retrospective observational study (5–9).

According to the International Classification of Headache Disorders, 3rd edition (11), TN is classified into classical, secondary, and idiopathic types. Classical TN, accounting for approximately 75% of all cases, is typically attributed to neurovascular compression. Secondary TN, comprising about 15% of cases, is caused by underlying pathological conditions such as brain tumors or demyelinating diseases, including multiple sclerosis (12). The remaining 10% include rare diseases such as familial TN (13).

In this case, persistent sensory abnormalities occurred after surgery, suggesting the possibility of concomitant post-traumatic painful trigeminal neuropathy (PPTTN). PPTTN arises from trigeminal nerve injury, leading to peripheral sensitization triggered by the activation of inflammatory mediators and immune cells, followed by central sensitization resulting in persistent chronic pain (14). However, in this case, the pain progressively worsened with compression of the trigeminal nerve due to tumor growth. Severe paroxysmal pain was elicited by stimulation of a trigger zone on the cheek, suggesting that the pain in this case was due to secondary trigeminal neuralgia. While coexistence of PPTTN is possible, it is unlikely to be the primary cause of the pain. The time course of this case also differs from that of PPTTN, which typically develops within six months of nerve injury (11).

Classical TN is typically caused by vascular compression of the trigeminal nerve at the root entry zone (REZ), most commonly by the superior cerebellar artery, anterior inferior cerebellar artery, vertebral artery, or venous structures. For classical TN, the first-line pharmacological treatment is CBZ and oxacarbazepine, a voltage-gated sodium channel blocker (1). According to the European Academy of Neurology guideline (1), there are insufficient data regarding pharmacotherapy for secondary TN, and treatment strategies similar to those for classical TN are recommended. Even in patients whose TN is well controlled with oral medications, acute exacerbations can occasionally occur, resulting in disabling pain and often requiring hospitalization. Paullet et al. have reported that over 90% of TN crisis cases are triggered by eating or speaking, and that about half of such patients experience difficulty drinking, two-thirds exhibit weight loss, and more than half show signs of anxiety or depression (3). Their study showed that secondary TN progressed to acute exacerbation more rapidly than classical TN, and was not associated with cranial autonomic symptoms such as lacrimation or conjunctival injection. However, the two TN types showed no significant differences in the duration of acute exacerbations or trigger factors.

No definitive acute-phase treatment for TN crisis has been established. Previous reports have described the application of local anesthetic injections to trigger zones, intranasal lidocaine spray, magnesium sulfate infusion, botulinum toxin injections, and subcutaneous sumatriptan (1, 2). Schnell et al. examined the efficacy of intravenous phenytoin and reported immediate pain relief in 89% of cases, although 15% showed side effects such as nystagmus, dysarthria, ataxia, or hypotension (4). Recently, several reports have described intravenous fosphenytoin as an effective and safer therapeutic alternative for exacerbation of classical, but not secondary TN (5–9).

The presumed pathophysiology of TN centers around focal demyelination of the trigeminal nerve at the REZ, most commonly caused by vascular compression (15–18). In the demyelinated regions, voltage-gated sodium channels at the nodes of Ranvier become ectopically expressed and functionally dysregulated (19, 20), leading to abnormal neuronal excitability (21). Damaged myelin allows ephaptic transmission, a short-circuiting phenomenon where excitation spreads to adjacent nerve fibers, propagating aberrant signals (22). This mechanism overlaps with that of allodynia, in which non-noxious stimuli provoke pain (23). Given these mechanisms, benign tumors such as meningiomas may induce demyelination and subsequent neuropathic pain through chronic compression of the trigeminal nerve. Accordingly, sodium channel blockers such as CBZ are considered effective in suppressing abnormal neuronal excitability in TN.

CBZ suppresses abnormal neuronal firing by blocking voltage-gated sodium channels, but is only available in oral form, making it unsuitable for patients unable to tolerate oral intake during acute TN exacerbations. Fosphenytoin, which shares a similar mechanism of action with CBZ, can be administered intravenously, providing rapid analgesia, and has emerged as a promising acute-phase therapy for TN.

Noro et al. reported that intravenous fosphenytoin significantly reduced pain within 24 h in patients with classical TN, regardless of prior surgery, nerve block, or radiosurgical intervention (5). In their study, the loading dose of fosphenytoin ranged from 9.8 to 20.7 mg/kg (750–1,200 mg per dose), followed by a maintenance dose of 7.5 to 9.5 mg/kg (422–750 mg per dose), and we adopted a dosing regimen consistent with this approach. Cheshire et al. described three patients with long-standing, medication-refractory TN who experienced complete pain relief after fosphenytoin administration (7). However, all of these studies focused solely on classical TN. To our knowledge, no previous reports have described the therapeutic efficacy of fosphenytoin for acute exacerbation of secondary TN. Our findings suggest that intravenous fosphenytoin would be a rapid and effective option for management of acute pain in secondary TN, potentially serving as a bridge to elective treatments including dose elevation, tumor removal or stereotactic radiosurgery.

## Conclusion

We have reported a patient with TN crisis due to petroclival meningioma who responded remarkably well to intravenous fosphenytoin. Intravenous fosphenytoin may be an effective treatment for acute pain relief in patients with TN crisis secondary to meningioma.

## Data Availability

The original contributions presented in the study are included in the article/Supplementary Material, further inquiries can be directed to the corresponding author/s.
